# Spatiotemporal Analysis of Malaria Transmission in the Autonomous Indigenous Regions of Panama, Central America, 2015–2022

**DOI:** 10.3390/tropicalmed9040090

**Published:** 2024-04-22

**Authors:** Alberto Cumbrera, José Eduardo Calzada, Luis Fernando Chaves, Lisbeth Amarilis Hurtado

**Affiliations:** 1 Dirección de Investigación y Desarrollo Tecnológico, Instituto Conmemorativo Gorgas de Estudios de la Salud, Panamá City 0816-02593, Panama; acumbrera@gorgas.gob.pa; 2 Departamento de Investigación en Parasitología, Instituto Conmemorativo Gorgas de Estudios de la Salud, Panamá City 0816-02593, Panama; jcalzada@gorgas.gob.pa; 3 Facultad de Medicina Veterinaria, Universidad de Panamá, Panamá City 0816-03366, Panama; 4 Department of Environmental and Occupational Health, School of Public Health, Indiana University, Bloomington, IN 47408, USA; 5 Department of Geography, Indiana University, Bloomington, IN 47408, USA; 6 Departamento de Análisis Epidemiológico y Bioestadística, Instituto Conmemorativo Gorgas de Estudios de la Salud, Panamá City 0816-02593, Panama; 7 Facultad de Ciencias Naturales y Exactas, Universidad de Panamá, Panamá City 0816-03366, Panama

**Keywords:** indigenous populations, human migration, geographical information science, malaria elimination, Panama

## Abstract

Despite ongoing efforts for elimination, malaria continues to be a major public health problem in the Republic of Panama. For effective elimination, it is key that malaria foci and areas of high transmission are identified in a timely manner. Here, we study malaria transmission records for the 2015–2022 period, a time when cases have increased by a factor of ten. Using several methods to study spatial and spatiotemporal malaria confirmed case clusters at the level of localities, including LISA and scan, we found that cases are clustered across indigenous villages located within the autonomous indigenous regions of Ngäbe–Buglé, Guna Yala, and Embera, with the latter on the eastern border of Panama (with Colombia). We discuss the different factors that might be shaping the marked increase in malaria transmission associated with these clusters, which include an inflow of malaria-exposed migrating populations hoping to reach the USA, insufficient health services, and the lack of culturally sensitive actionable tools to reduce malaria exposure among the ethnically diverse and impoverished indigenous populations of Panama.

## 1. Introduction

**Figure 1:**
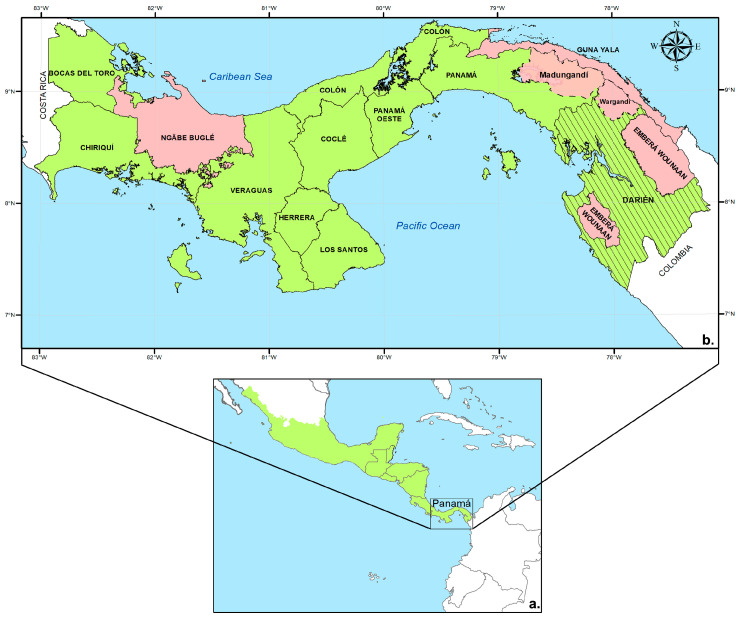
Republic of Panama: (**a**) location within the Mesoamerican region; (**b**) distribution of malaria-endemic areas. The indigenous autonomous regions (comarcas) are marked in pink and the striped areas correspond to the province of Darien, home to several indigenous communities.

Panama, located in the southernmost part of the Mesoamerican subregion (Figure 1), has been identified by the World Health Organization (WHO) as a candidate for the elimination of malaria by 2025 [1]. To achieve this nationwide goal, in 2017, the country launched a National Malaria Elimination Programme (NMEP) [2].

**Figure 2:**
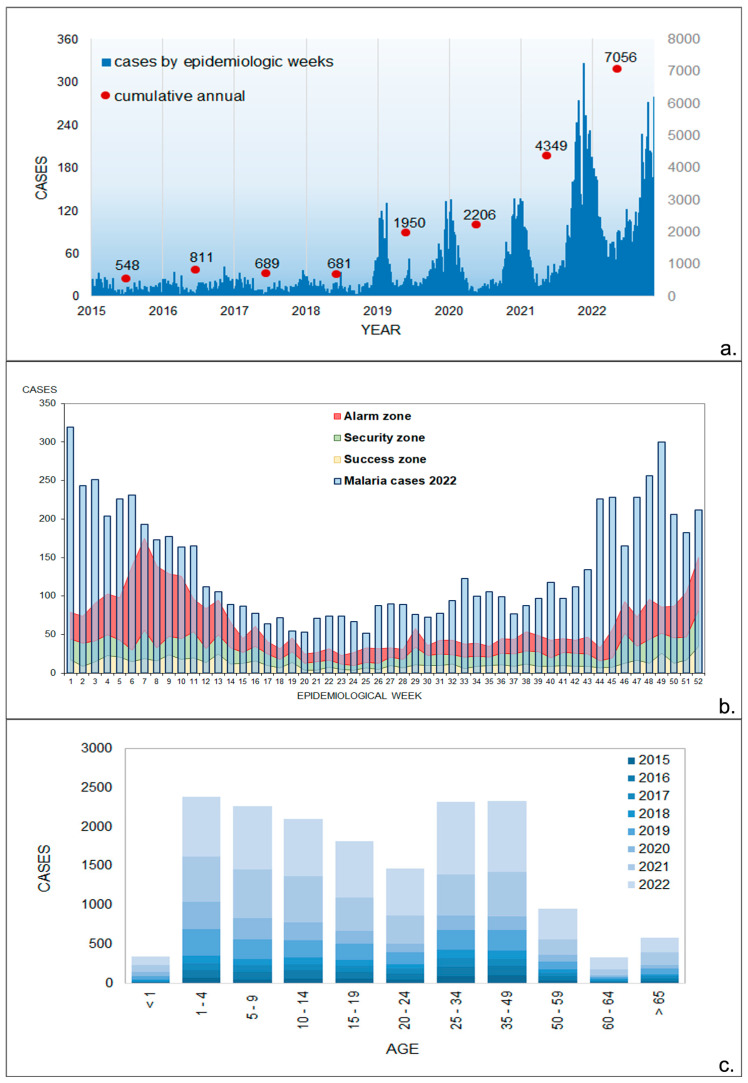
Malaria in Panama from 2015 to 2022: (**a**) number of cases by epidemiologic week and year; (**b**) endemic channel (2015–2021) constructed using the quartile method and the weekly incidence recorded in 2022; (**c**) malaria cases by age group.

During the last two decades, however, malaria transmission intensity and infection risk in the country have shown significant spatial and temporal fluctuations, with periods of reduced transmission followed by periods of large epidemics (Figure 2a). Many abiotic and biotic factors have been suggested as responsible for these frequent malaria resurgence events, including cross-cultural barriers to control efforts in endemic areas, El Niño Southern Oscillation (ENSO) and other climatic fluctuations, parasite/vector resistance and an increase in imported malaria cases [3]. However, behind most of these resurgence events, a clear weakening of the malaria control program has hindered the prompt detection of increases in malaria transmission and the implementation of timely and effective control measures [3].

At present, precisely when Panama is geared towards elimination, the situation is similar to what was recorded back in the 1950s [3]. In fact, since 2017 when the national elimination program started, malaria cases have been steadily increasing from 683 malaria cases in 2017 to 7112 in 2022; representing a 10.4-fold increase (Figure 2). Moreover, *Plasmodium falciparum* transmission, considered to be virtually eliminated in the country, has been re-established in eastern regions of the country, and transmission has spread to other regions where malaria has been absent for decades [4].

Undoubtedly, the COVID-19 pandemic negatively impacted malaria elimination efforts and previous achievements [5], mainly because of human and financial resource diversion to tackle the pandemic [5,6,7]. During this period, indoor insecticide spraying cycles, as well as other vector control activities that were planned in the NMEP guidelines, were almost completely interrupted, and until now serious disruptions in the program persist.

Under this scenario, characterized by a shortage of human/financial resources and widespread malaria transmission, the NMEP must rethink the most efficient and cost-effective approaches to reduce malaria transmission and approach malaria elimination. One way to efficiently approach the national elimination efforts is to initially concentrate control measures and interventions in high transmission or highly vulnerable areas. To identify these areas and consequently allocate resources and efforts, spatial and spatiotemporal cluster analyses are needed, preferably with an operational high geographical resolution.

A previous spatial study carried out in Panama from 2000 to 2014 identified spatial foci of increased transmission in the country [8]. However, that study was performed years before the NMEP was initiated and at a low spatial resolution. Since then, the malaria epidemiological situation in Panama has changed significantly, not only in its spatial distribution but also in transmission intensity. In this context, the objective of this study was to quantify the spatiotemporal distribution of malaria in the country, mapping epidemiological data at the level of localities. We also evaluated environmental indicators associated with active transmission foci.

## 2. Materials and Methods

### 2.1. Study Area

A descriptive and ecological study applying Geographic Information Science (GIS) and statistical tools was conducted in Panama, a country situated at the southeastern end of the Central American isthmus (Figure 1). Panama is within the intertropical zone near the equator, between coordinates 7°12′07″ and 9°38′46″ north latitude and 77°09′24″ and 83°03′07″ west longitude [9]. It has an area of 75,517 km^2^, with 65.4% of its land covered by forests [9,10]. It is limited to the north with the Caribbean Sea, to the south with the Pacific Ocean, to the west with Costa Rica, and to the east with Colombia, forming a biological corridor that joins South America with the rest of Central America.

During the past few decades, malaria transmission in Panama has been clustered across the autonomous indigenous regions, or “comarcas”, of the country. The comarcas are mainly inhabited by indigenous populations. The comarcas include Madungandí, Wargandí, Emberá–Wounáan, and Guna Yala in eastern Panama, and Ngäbe–Buglé in western Panama (Figure 1b). Malaria cases in these comarcas represent about 90 percent of all diagnosed cases in the country [3]. Altogether, the “comarcas” occupy 22.2% of the country’s area, and their estimated 698.114 inhabitants make up 17.2% of the country’s population [9].

The poverty rate in the indigenous population of Panama (82%) is four times higher than that of the country as a whole (20.7%). Indigenous populations also have higher rates of multidimensional poverty, meaning that beyond a lower income, they also have less access to services and opportunities to improve their socio-economic conditions [11]. The scarce health infrastructure (including lack of health personnel and access to medicines and technological supplies), economic and geographic barriers, and the weak intercultural approach to addressing health issues in the “comarcas” have increased access barriers to health services by the Panamanian indigenous population [11].

The intertropical zone where Panama is geographically located is isothermal, with little temperature variation throughout the year (2 °C to 5 °C). The temperature generally ranges from 24 °C to 32 °C. Panama has a tropical rainforest climate with a so-called dry season, characterized by an almost total absence of rain from January to March. The rainy season spans from May to November. April and December are considered transition months. Humidity is high throughout the year in most of the country, but during the rainy season, it can reach almost 100% [12].

The western Caribbean region, where the malaria-endemic Ngäbe–Buglé comarca is located, is the rainiest in the country, with around 261 rainy days per year and no low-rain (dry) season. On the other hand, Guna Yala in the eastern Caribbean is one of the least rainy regions in the country with a maximum of 85 rainy days per year. Meanwhile, the Comarcas Madungandí, Wargandí, and Emberá–Wounáan, located in the eastern Pacific region, have a distinctly lower rainy period from January to March [12,13].

### 2.2. Data Collection and Sources

Malaria cases in Panama are mostly detected by active surveillance performed by the National Malaria Control Programme personnel in all endemic areas. Cases are confirmed by microscopy of Giemsa-stained smears or by rapid diagnostic tests applied in remote areas [4]. Epidemiological anonymized data on malaria confirmed cases by species were provided by the Ministry of Health. Information included in this study were epidemiological week, age, and gender of each case registered from 2015 to 2022. Geographic and related data were obtained from the database of the Vector Control Department, Ministry of Health. Collected data included the exact geographic origin of confirmed cases by province, district, corregimiento, and locality (the four levels of administrative units in Panama). Data regarding the population and cartography of provinces and populated places were provided by the National Institute of Statistics and Census [9]. The health facility locations in Panama were obtained with the hosted Feature Layer called “health infrastructures”, which is shared by the Ministry of Health using an ArcGIS Online platform for public use. This license is currently held by the Gorgas Memorial Institute for Health Studies. The altitude of each location was determined using the Google Earth Pro program.

Climatic variables (1981–2022) were obtained through NASA’s Prediction of Worldwide Energy Resources (POWER) project, which was created to improve the current renewable energy dataset and create new environmental datasets from new satellite systems. We used the Data Access Viewer at https://power.larc.nasa.gov/data-access-viewer/, (accessed on 2 January 2024) which allowed downloading climate variables over time for each location.

### 2.3. Data Analysis

#### 2.3.1. Descriptive Analysis

Descriptive analyses were performed on confirmed malaria cases by epidemiological week, age, gender, and seasonal pattern (monthly cases), represented in the form of graphs, boxplots, and maps. An endemic channel was created using the quartile method with weekly malaria case data spanning from 2015 to 2022 [14]. The quartiles for the data reported by epidemiological week between 2015 and 2021 were calculated, including Q1 (25th percentile, representing the success zone), Q2 (median, indicating the safe zone), and Q3 (75th percentile, representing the alarm zone). Based on these percentile values and the malaria cases reported in the different epidemiological weeks of 2022, a stacked line and bar graph were generated, respectively. The accumulated cases in each epidemiological week that exceeded the limit of the alarm zone were considered to be epidemics. As a result, we examined case frequency in each epidemiological week based on the zone they belong to.

An ANOVA was conducted to compare the mean number of malaria cases across various age groups and to assess whether there was a statistically significant difference between age groups or if any observed differences were mere chance occurrences. This test evaluates the variation between groups and within each group using the F-test. If the variance between groups exceeds the variance within groups, it is concluded that the means of the groups are unequal [15]. The calculation of effect size (η^2^) was included, which establishes the percentage of the dependent variable (cases) that can be explained by the independent variable (age groups). An η^2^ around 0.01 is usually considered to be a small effect, an η^2^ around 0.04 indicates a medium effect and an η^2^ above 0.1 is a large effect. The Student *t*-test was used to compare the average number of confirmed cases between genders [15].

To analyze the seasonality of malaria cases, we evaluated the case distribution for every month and county between 2015 and 2022 using box plots. The inner line in the plot presents the median number of cases (second quartile), while the ends of the boxes represent the first (lower) and third (upper) quartiles. Additionally, whiskers extend from each quartile towards the lowest and highest values of the case distribution. Each whisker indicates the variability outside the lower and upper quartiles, respectively [15].

A principal component analysis (PCA) was used to describe the relationship between some of the determinants of malaria transmission in localities with more than 100 cases during the studied period. Only those localities with high cumulative transmission for more than four years were considered in the analysis. The set of covariates studied included the following: temperature at 2 m (°C) = Temp; minimum temperature at 2 m (°C) = Temin; maximum temperature at 2 m (°C) = Temax; relative humidity at 2 m (%) = Humedad; rainfall at 2 m (mm) = Precip; wind speed at 10 m (m/s) = Wind; distance to the assigned health facility (m) = Healthfa; and altitude (m) = Alti. The PCA is considered a technique that evaluates the existing relationships between a set of variables. The analysis began with the normalization of the variables as their scales of measurement were very different. Then, we proceed with the evaluation of correlations between variables, from the correlation matrix. With this matrix, it was possible to determine the eigenvectors (direction of the principal component) and the eigenvalues (variance of the principal components), and, with this information, the sequence of principal components (or axes) was formed. A principal component, a new variable, was then created by the linear combination of the original variables. In this study, the criterion used to select the principal components was to keep those with an eigenvalue greater than 1 (Kaiser Criterion) [16]. Meanwhile, the quality of the representation of the covariables was determined using a cosine square plot. The closer a variable is to the circle of correlations, the more representative it is considered.

ArcGIS Desktop 10.7 geoprocessing tool was used to create the shapefile of distances to the nearest health center (km), altitude (msnm), and distance to water bodies for each locality with more than 100 cases.

#### 2.3.2. Spatial Analysis

##### Spatial Reference Matrix

To implement the analyses described in the following subsections, a spatial reference matrix (georeferencing) was created by locating each populated place with its coordinates (longitude and latitude) using a shapefile of populated places provided by the Instituto Nacional de Estadística y Censo (INEC) [9].

##### Spatial Autocorrelation

Cluster and outlier analysis (Anselin Local Moran’s I) detects when a feature is surrounded by features with similarly high or similarly low values [17]. In this case, this entity is part of a cluster. A negative value for I indicates that a feature has neighboring features with different values, meaning that this entity is an outlier. Five possible categories are classified by Moran’s I index: high–high, low–low, high–low, low–high, and not significant. High–high locations indicated hot spots for malaria incidence, while low–low locations indicated cold spots. The high–low and low–high areas were considered outliers. The cluster/outlier type (COType) field distinguishes between a statistically significant cluster of high values (high–high), a cluster of low values (low–low), an outlier in which a high value is surrounded primarily by low values (high–low), and an outlier in which a low value is surrounded primarily by high values (low–high).

##### Spatial Clusters and Spatiotemporal Clusters Based on the Scan Method

A purely spatial scan analysis was used to detect and analyze spatial clusters of malaria cases regardless of time. The purely spatial scan statistical analysis method consists of generating numerous overlapping circular windows that scan the study area. The radius of the circular window varies in a range from 0 to a preset maximum size, which refers to the maximum percentage of the population at risk. Each window is considered a possible cluster. In this study, the maximum cluster size was specified as 50% of the population at risk of malaria transmission. The null hypothesis is that the risk of malaria transmission is the same inside and outside the circular window in space, while the alternative hypothesis is that the risk of transmission inside the circular window is different from that outside the circular window. For each window, the number of observed malaria cases both inside and outside the circle is counted and compared with the expected malaria cases, on the basis that they follow a Poisson distribution. Under this information, the likelihood ratio within each window is obtained. The circular window with the maximum likelihood ratio at the same time with more cases than expected is marked as the most likely group that is least likely to have occurred at random [18]. The likelihood ratio for this circular window comprises the maximum likelihood ratio test statistic. The *p*-value was estimated using 999 Monte Carlo simulations.

The scan method was also used to identify spatiotemporal clusters with high (and low) malaria incidence and to test their statistical significance. We used the discrete Poisson model given the count nature of our data. The scan method for spatiotemporal analysis consists of generating numerous cylindrical windows with a circular or elliptical base of a specific size, overlapping, which scan together the total study area to detect clusters with the highest likelihood ratio. Given the geometry of Panama, which is more linear than planar, we used elliptic search windows with SaTScan [19]. The base of the cylinder is the cluster area, and the height reflects the temporal scanning window [20]. The maximum size specifies the largest percentage of the total population at risk within the window. In this study, the maximum window size was set at 50% of the population at risk. Each window is considered a possible candidate group and the one with the maximum log-likelihood ratio (LLR) and the highest number of expected cases is identified as the most likely cluster. The remaining (secondary) clusters are then ranked successively according to the LRR value. The relative risk (RR) represents the risk within the window compared to the risk outside the window0. The *p*-value was estimated using 999 Monte Carlo simulations. A significance level of alpha < 0.05 was used to test whether or not the clustering was significant. 

### 2.4. Software and Packages

The following software was used to perform statistical analyses: R software version 4.2.3, ArcGIS Desktop version 10.7 (ESRI Inc., Redlands, CA, USA), Google Earth Pro version 7.3.6.9796 (64-bit), ArcGIS version Online, POWER Data version 2.0.0, and Microsoft Excel version 2013. Scan statistics were estimated using the SaTScan^TM^ software (V 10.1) developed by Kulldorff [18,20].

## 3. Results

### 3.1. Epidemiological Data

A total of 17,063 confirmed malaria cases were registered in Panama between 2015 and 2022 (Figure 2a). In the last three years evaluated in this study (2020, 2021, and 2022), the number of malaria cases was four times higher when compared with the four initial years (2015 to 2018) (3563 vs. 13,500). In 2022, the total number of malaria cases (7112) reached a peak in the 65-year history of malaria recorded by malaria programs in Panama. In fact, in 2022, there was an unexpected rise in the weekly count of infected individuals with malaria cases, remaining above the epidemic threshold in all the epidemiological weeks. This occurrence was observed in accordance with the historical context of the cases (Figure 2b). The age of confirmed malaria cases was classified into five-year age groups. The data indicate that malaria was prevalent in all age groups (Figure 2c). In the groups investigated, malarial transmission occurred in 2383 (16%) children between 1 and 4 years of age, which was the most affected group. This was followed by the age groups 25 to 29 and 5 to 9 years, with 2318 (15%) and 2268 (15%) cases reported, respectively. There was a statistically significant difference (*p* = 0.012) in the number of cases recorded among the various age groups analyzed, with a large effect size (η^2^ = 0.24). In contrast, when evaluating the average number of cases considering gender, we did not observe a significant difference (*p* = 0.456).

### 3.2. Spatial Distribution of Malaria Cases in Endemic Areas

**Figure 3:**
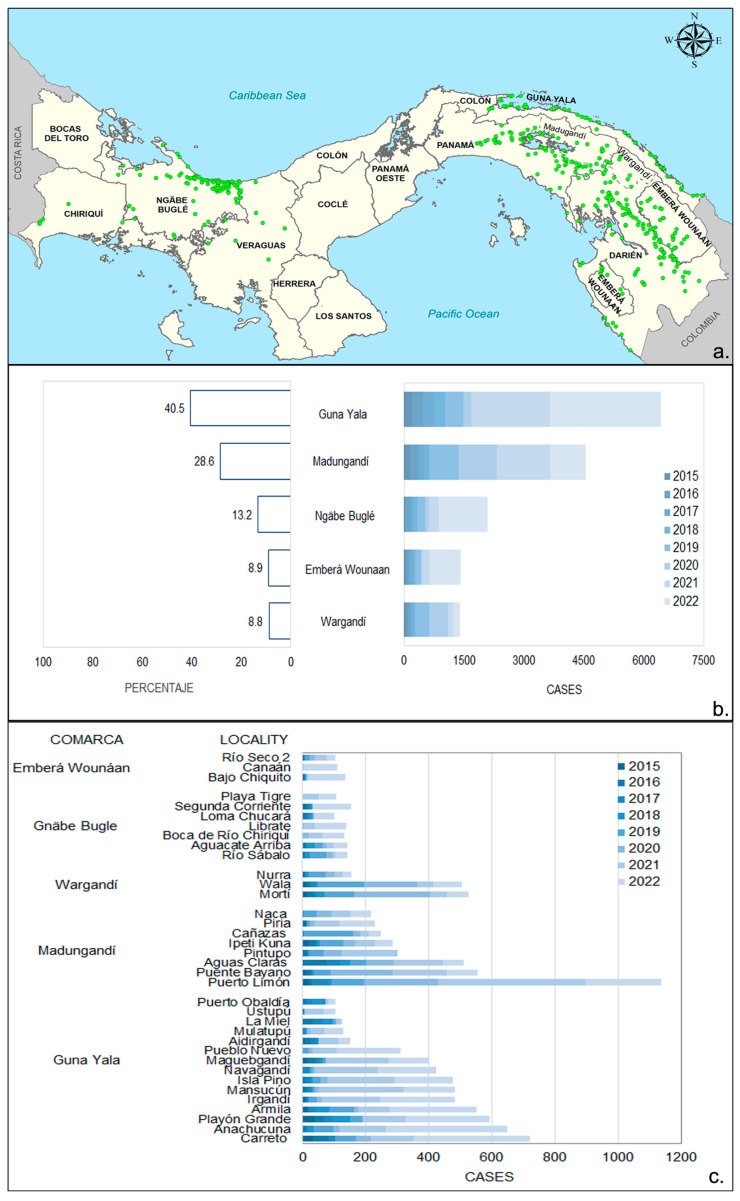
Malaria in the indigenous comarcas 2015–2022: (**a**) spatial distribution of localities with at least one confirmed case; (**b**) distribution and annual percentage of malaria cases within the comarcas; (**c**) indigenous localities with more than 100 accumulated cases. For further details about transmission intensity, please refer to the data analysis section in the methods.

Figure 3a shows the spatial distribution of localities with malaria case records in Panama from 2015 to 2022. During this eight-year period, there were reports of at least 1 malaria case in 404 localities. Of these, 226 localities were strictly situated within indigenous comarcas and were distributed as follows: 55% (124/226) belonged to the comarcas located in the eastern region of the country, mostly inhabited by Guna populations, and accounted for 80% (12,373/15,884) of all malaria cases observed during this period (Figure 3b). In the western region of the country, the Ngäbe–Buglé ethnic group comprised 13.2% (2093/15,884) of the malaria cases observed during this period (Figure 3b). In total, 36 localities within the comarcas had more than 100 cumulative cases (Figure 3c).

Of the 183 (46%) localities with malaria records located outside the comarcas, 63 (34%) were from the eastern side of the Panama province. Most of them are communities bordering Madungandí comarca, and 100 (57%) were from populated places in Darién near the Colombian border. In the western part of the country, the localities with cases outside the comarcas belong to Veraguas (14; 7.6%) and Chiriqui (6; 3.7%). It is important to emphasize that Ngäbe–Buglé communities inhabit and are a majority in localities from the entire Ngäbe–Buglé comarca, as well as nearby areas included in the bordering provinces (Chiriquí and Veraguas).

### 3.3. Monthly Malaria Cases

The weekly malaria cases time series shows more accurately how the endemic pattern of malaria in the different regions has been highly variable over recent years. Since 2019, a shift in transmission dynamics has been observed (Figure S1). In the comarcas Ngäbe–Buglé and Guna Yala, the main outbreak comprised around 30 cases, while in Madungandí, it exceeded 60. When analyzing the annual evolution of malaria between the comarcas, it is evident that the situation of malaria in Panama largely reflects malaria transmission in Madungandí (Figure 2a and Figure S1).

Malaria transmission patterns in the comarcas might reflect differences in the climate and environmental parameters. For example, seasonality was very similar in Madungandí and Wargandí, which are comarcas located in the eastern Pacific region. Indigenous localities from Madungandí (2007/3673; 55%) and Wargandí (1180/1824; 65%) accumulated the highest number of cases between January and March when rainfall in that region of the country is less frequent. By contrast, no defined seasonality in monthly malaria transmission patterns was observed in Ngäbe–Buglé (Western Caribbean region) and Guna Yala (eastern Caribbean region). However, in Ngäbe–Buglé comarca, 41% (484/1359) of the malaria cases confirmed were reported between July and September. In the other comarcas, malaria outbreaks are frequent in the first quarter of the year, with a pronounced increase in outliers occurring from April to November.

### 3.4. Trends in Environmental Factors

Between 1981 and 2021, locations with high malaria transmission (>100 accumulated cases) exhibited a maximum temperature range of 28.58 °C to 31.04 °C and a minimum temperature range of 20.88 °C to 26.10 °C. The average temperature varied from 25.39 °C to 27.34 °C, while relative humidity values ranged between 81.29% and 87.82%. The average rainfall varied between 149.42 mm and 251.60 mm. In these high transmission communities, the distance to a healthcare facility ranged from 0 to 56 km, but for 50% of the areas, it exceeded 16.5 km. The elevation range of the localities was wide and variable, with localities having altitudes between 3 and 195 m. Coastal communities in Guna Yala have the lowest altitudes among the studied localities.

**Figure 4:**
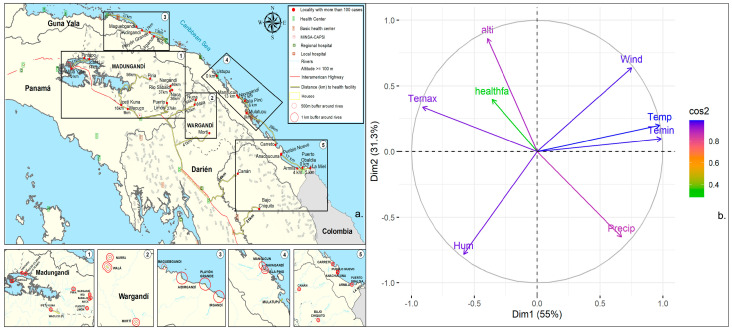
Cumulative malaria cases between 2015 and 2022 in areas situated in the country’s eastern region with high malaria transmission (above 100 cases): (**a**) geographical location; (**b**) a correlation circle detailing the contribution of determinants of transmission to each principal component. A high value in the circle implies a good representation of the variable in that component (color-coded blue). Temperature at 2 m (°C) = Temp; minimum temperature at 2 m (°C) = Temin; maximum temperature at 2 m (°C) = Temax; relative humidity at 2 m (%) = Hum; rainfall at 2 m (mm) = Precip; wind speed at 10 m (m/s) = Wind; distance to the assigned health facility (m) = Healthfa; and altitude (m) = Alti.

The PCA suggests that malaria cases are more closely associated with maximum temperature and altitude. Associations were also positive with all other temperature measurements, but negative with relative humidity and rainfall. Rainfall was the least associated variable with changes in malaria cases (Figure 4b).

### 3.5. Spatial Analysis

#### 3.5.1. LISA Cluster Analysis

**Figure 5:**
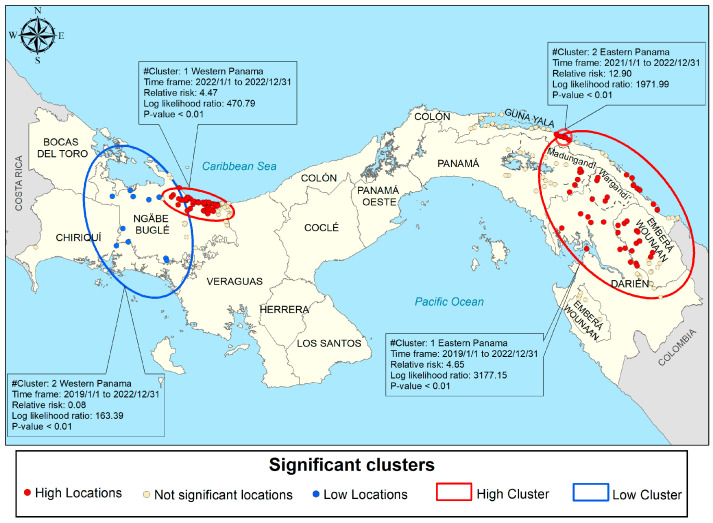
Spatial distribution of detected space–time malaria clusters from 2015 to 2022. Each ellipse represents a cluster in the spatiotemporal analysis.

Risk assessment considering neighboring localities showed that in Panama there has been a spatial autocorrelation in malaria cases during the study period. The sequence of years in Figure S3 detected the existence of localities in a high-risk condition (HH cluster). In particular, the detection of these clusters was notable in the Guna Yala comarca (14 HH localities). An exceptional case occurred in 2019 in which, under an increase in transmission between nearby localities belonging to the comarcas of Madungandí and Wargandí, eight clusters emerged in a high transmission situation (Figure 5 and Figure S2). On the other hand, in Ngäbe–Buglé localities, most spatial clusters occurred in 2016. Between 2020 and 2021, years with mobility restrictions due to the pandemic, low transmission (LL) clusters stand out. Some of these clusters belonged to the indigenous reservation formed by the Emberá and Wounáan comarca. Meanwhile, in the localities bordering the Republic of Colombia, variability in risk status was observed year after year.

#### 3.5.2. Purely Spatial Scan Cluster Analysis

With the purely spatial analysis model, the existence of spatial clusters was detected in the eastern and western regions of the country, mainly after 2019 (Figure S3). Unlike the clusters located in the east of the country, the area where the most probable clusters persistently occurred was in the west, and the number of localities within the elliptical window has increased for western Panama.

#### 3.5.3. Spatiotemporal Scan Cluster Analysis

Spatiotemporal scan statistics were used to detect high-risk clusters of malaria infection from 2015 to 2022 (Figure 5). Two spatiotemporal malaria clusters were detected in the comarcas located in the eastern part of Panama. The first (most likely) cluster comprised 49 localities with 10,639 people at risk from both the Guna and Emberá comarcas, spanning from January 2019 to December 2022, with a RR = 4.65 (LLR = 317,715, *p* < 0.01). Between January 2021 and December 2022, a second cluster comprised a population of 967 inhabitants from seven localities in Guna Yala, with a RR of 12.9 (LLR = 1971.99; *p* < 0.01). These clusters are consistent with the season of greatest complexity in the epidemiology of malaria in the country, i.e., a time when cases were increasing between 2019 and 2022, corresponding to epidemic years (Figure 2). Meanwhile, in the west, a cluster was found covering 50 northern Ngäbe–Buglé localities where 1408 individuals were at risk, with a RR = 4.47 (LLR = 470.79, *p* < 0.01) for the year 2022. Between 2019 and 2022 a low transmission cluster was found in southern Ngäbe–Buglé localities (Figure 5).

## 4. Discussion

In this study, a combination of GIS and geospatial techniques was used to identify clusters of malaria cases between 2015 and 2022 at the community level in Panama. The analysis of the spatial clusters showed that malaria was not randomly distributed in the country, but clustered in the autonomous indigenous territories. Although specific communities that were included in significant clusters varied over the years studied, the areas of high malaria transmission were generally the same. This scenario may arise because of implementing immediate control measures in a specific location without simultaneously targeting neighboring communities. This issue is frequently observed across the country, often attributed to personnel shortages and limited resource availability. Nevertheless, differences in the intensities and distribution of significant clusters were observed at different time periods. For instance, at the beginning of the National Malaria Elimination Programme in 2017–2018, there was an important decrease in malaria significant clusters compared with 2015 and 2016. Since 2019, however, the number and magnitude of malaria significant high clusters progressively increase to reach a maximum in 2022 (Figure 5). Furthermore, since 2019, the number of significant clusters also increased in the western region of the country, a trend not observed in preceding years. Unfortunately, data for 2023 were not incorporated into this study. However, it is noteworthy that malaria cases surged significantly from 2022 to 2023, with reported cases escalating from 7112 to 11,057, a 65% increase.

Despite its small size and narrow shape, malaria eco-epidemiology in the eastern side of Panama differs substantially from the one observed in the western side. For instance, while *P. vivax* remains the predominant malaria species on both sides, constituting over 95% of reported malaria cases in the country since 2000 [3], transmission of *P. falciparum* has only been re-established periodically over the past two decades in various indigenous communities situated in the eastern region. Recent molecular studies have shown that *P. falciparum* parasites circulating in this eastern region are resistant to chloroquine and partially resistant to antifolates [21,22,23], a resistant profile not observed in *P. falciparum* parasites circulating in the rest of the Mesoamerican countries. It has also been described that malaria parasites from the eastern side have a higher genetic diversity [24,25], as well as a higher vector species richness [26]. The ecological conditions also differed, particularly in the amount and seasonal distribution of rainfall, mean annual temperatures, daily fluctuations in temperature, and landcover [27]. In fact, our PCA analysis suggested that malaria cases were more associated with maximum temperature and altitude; in general, eastern Panama is hotter than western Panama, where average elevation is higher in the latter (Figure 4b). This heterogeneity suggests that parasite/vector biological characteristics, as well as the environmental risk factors prevalent in the western and eastern comarcas of Panama, might have influenced local malaria transmission and, consequently, affected the observed clustering in the country. However, biologically, since the temperature is very important for transmission, the higher and near-optimal conditions for parasite development might have rendered eastern Panama more prone to malaria outbreaks. Nevertheless, on top of these malaria transmission drivers, other factors such as the uncontrolled migration through the permeable Colombian–Panamanian border and the high mobility of the indigenous population may be the main triggers for the spread of malaria in the country. In 2023, Panama registered a record number of 520,085 irregular migrants who entered the country through the Darién Gap (more than double compared to 2022), aiming to reach the United States or Canada [28]. An estimated 20% are children under five years old. Most migrants crossing the Darién gap are from the South American and Caribbean countries of Venezuela, Haiti, and Ecuador, but some are from as far as sub-Saharan Africa, the Middle East, and Asia [28].

For multiple reasons, including poverty, violence, political instability, and climate change impacts, northward migration has been increasing [29]. In Venezuela, the Latin American country with the highest malaria incidence since 2018 [30], migration has accelerated after the economic sanctions from the USA and the extremely high inflation observed in that country [31]. Linked to unregulated migration is the escalating incidence of imported malaria cases documented in Panama over recent years, which poses a significant hurdle to the National Malaria Elimination Program goals [3]. The majority of migrants moving northward originate from regions where *Plasmodium* spp. exhibit resistance to chloroquine, potentially serving as the reservoir for the introduction and subsequent dissemination of drug-resistant parasites to currently deployed antimalarial medications in the area [32]. For instance, local cases of *P. vivax* malaria recently identified in the Southern United States may be linked to this migratory influx [33]. Primarily influenced by their economic status and prevailing weather conditions, northward migrants can arrive in Panama from Colombia via various routes, with the majority trekking through the Darien Gap [34]. Upon reaching Panama, their migratory journey typically involves passage through numerous indigenous transit communities, many of which are recognized as hotspots for malaria transmission situated within significant malaria clusters. For instance, Carreto serves as a significant entry point situated in the eastern Kuna Yala comarca and has persistently served as a hotspot for malaria transmission within the country (Figure 5), where chloroquine-resistant *P. falciparum* parasites have been identified [21]. Additionally, Puerto Limón in the eastern Madungandi comarca has emerged as a novel arrival point for irregular migrants en route to the United States. While this community has experienced reduced transmission rates in recent years, there has been a resurgence in the number of malaria cases and clusters at risk (Figure 5). Numerous other communities primarily inhabited by Guna populations, including Puerto Obaldia, La Miel, Armila, and Aswemullu, which hold key positions along the migratory route, have also been identified as hotspots once again since 2021 (Figure 5 and Table S2). It would be interesting to assess whether the surge in migrant arrivals to these communities could be linked to the rise in malaria transmission rates. There are high levels of connections and bonds among Guna populations living in different communities, whether within the same comarca or between distant Guna comarcas. These populations frequently move along trails or through various water sources for different reasons, mainly involving family visits, commercial activities or cultural events, such as frequent exchanges during Guna spiritual conventions. In fact, the high mobility of the Guna population is considered one of the main causes of the spread and maintenance of malaria transmission in this indigenous population [35]. For example, Gunas residing in Mortí, Nurra, and Wala in the comarca Wargandí are the primary source of malaria cases in the province of Darien. Their incidence rate in 2019 and 2020 placed the province in a high-risk cluster.

The indigenous inhabitants of the Ngäbe–Buglé comarca, on the western side of the country, have the lowest rate of health service use in the country, but still, there is an increase in notifications of positive malaria cases and high-risk clusters, especially in 2021 and 2022 (Figure 5 and Table S1). Ngäbe populations seasonally move to work in the coffee harvest in Costa Rica, settling in cities near the areas where coffee is cultivated. This seasonal migration occurs through Ngabe ancestral lands; indeed, most members from this ethnic group are binational (citizens of both Costa Rica and Panama) and commonly use health services in Costa Rica, especially in human settlements located in areas that are part of different routes that migrants use on their way to the USA and Canada. This situation highlights the importance of setting effective and coordinated cross-border malaria control initiatives, particularly when highly mobile populations are involved [36]. No clusters of malaria transmission were observed in the central area of Panama. This central region, the most urbanized and densely populated in the country, has been free of local malaria transmission for many decades. It has been described that the urbanization process results in significant socio-economic and landscape changes that typically reduce malaria transmission [37]. However, it also presents challenges associated with high levels of human mobility. As people infected with malaria migrate into urban and suburban settings, they can strain local health systems and can also contribute to sustained malaria transmission within certain urban communities.

Regarding the impact of the COVID-19 pandemic, it is important to consider that Panama was one of the countries in the region with the strictest lockdowns and containment strategies to prevent and mitigate the spread of the disease. These measures included border closures, travel restrictions, mandatory quarantines, mask mandates, social distancing protocols, selective closures, and a phased reopening plan. These measures were effective in reducing COVID-19 transmission and associated fatalities; however, they also had a detrimental impact on ongoing and planned malaria control activities at various levels. Activities affected by the “new normality” included the distribution of long-lasting insecticide-treated nets and indoor residual spraying of insecticide, which are conducted through house-to-house visits. Other important control measures, such as the use of rapid diagnostic tests and blood smear diagnostics and appropriate treatment of malaria, were also significantly disrupted, particularly in endemic regions in Panama where malaria control heavily relies on active surveillance and timely interventions.

While this study effectively identified spatial and spatiotemporal clusters of malaria using data spanning from 2015 to 2022, several limitations must be acknowledged. Firstly, the retrospective nature of these data could have impacted the study results due to potential issues with data quality. Many developing nations experience challenges such as incomplete reporting of routine data, non-reporting, missing data, and deficiencies in data aggregation frameworks. Regarding this matter, it is pertinent to mention the issue of record duplication in malaria studies. Duplication can occur when the same patient undergoes multiple tests to determine a cure, recrudescence, or relapse. This phenomenon can lead to inflated case counts and affect the accuracy of epidemiological data analysis. However, it is worth noting that malaria case management and data quality in Panama have shown significant improvement in recent years, largely attributed to collaborations with international global health organizations, particularly by the Clinton Health Access Initiative.

Another probable limitation of this study is the insufficient investigation of malaria cases, which may hinder the reliable differentiation between local and imported cases. While it is generally assumed that imported cases can be easily distinguished based on travel history, in some instances this may not hold true. It is crucial to make this distinction, especially considering the observation that many high-incidence malaria clusters tend to concentrate along migrant transit routes. Therefore, imported cases could have impacted the size and location of the high rates of malaria clusters detected in this study. Also, when evaluating highly mobile populations, the precise determination of the locality where individuals were initially infected, as opposed to the locality where they were captured and diagnosed, poses a considerable challenge. This misallocation of malaria cases to specific localities can significantly impact the accuracy of our analysis.

## 5. Conclusions

This study analyzed malaria case data over eight years (2015–2022) at a fine geographic scale, focusing on localities. This approach yielded valuable insights into the spatial persistence of clusters within specific geographic regions of Panama. This integrated study offers novel insights into malaria transmission patterns, not only in space but also in time. The findings of this study confirmed previous observations indicating that high-risk areas for malaria are concentrated within indigenous comarcas on both sides of Panama. These results could serve as a basis for planning and implementing effective malaria control strategies. Moreover, our robust cluster identification, using several methods, can help inform optimal resource allocation by prioritizing regions with the greatest need, thereby maximizing the impact of interventions aimed at eliminating malaria transmission from Panama.

## Data Availability

Aggregate and anonymized data analyzed during this study are available from the corresponding author upon reasonable request and with permission from the Ministerio de Salud de la República de Panamá.

## Supplementary Materials

The following supporting information can be downloaded at: https://www.mdpi.com/article/10.3390/tropicalmed9040090/s1, Figure S1: weekly evolution and malaria transmission seasonality in each comarcas; Figure S2: spatial autocorrelation of malaria cases from 2015 to 2022; Figure S3: cluster of malaria cases detected using the purely temporal clustering from 2015 to 2022 in Panamá. Table S1: spatial scan statistics of significant clusters of malaria cases at the Indigenous Comarcas in the western region of Panama, January 2015 to December 2022; Table S2: spatial scan statistics of significant clusters of malaria cases at the Indigenous Comarcas in the eastern region of Panama, January 2015 to December 2022.

## References

[1] World Health Organization World Malaria Day: WHO Launches Effort to Stamp out Malaria in 25 More Countries by 2025. https://www.who.int/news/item/21-04-2021-world-malaria-day-who-launches-effort-to-stamp-out-malaria-in-25-more-countries-by-2025.

[2] Ministerio de Salud (2018). Plan Estratégico de Eliminación de la Malaria (PEEM) en Panamá 2018–2022.

[3] Hurtado L., Cumbrera A., Rigg C., Perea M., Santamaría A.M., Chaves L.F., Moreno D., Romero L., Lasso J., Caceres L. (2020). Long-term transmission patterns and public health policies leading to malaria elimination in Panamá. Malar. J..

[4] Ministerio de Salud, Dirección General de Salud Pública, Departamento de Control de Vectores, Departamento de Epidemiología (2023). Plan de Acción Rápida Para el Combate Contra la Malaria en focos Específicos de Áreas de Difícil Acceso.

[5] Loaiza Rodríguez J.d.R., Eastwood G., Chaves Sanabria L.F. (2023). Planetary Health Approaches to Understand and Control Vector-Borne Diseases.

[6] World Health Organization (2022). World Malaria Report 2022.

[7] The Commonwealth Malaria Report 2022. https://reliefweb.int/report/world/commonwealth-malaria-report-2022.

[8] Lainhart W., Dutari L.C., Rovira J.R., Sucupira I.M., Póvoa M.M., Conn J.E., Loaiza J.R. (2016). Epidemic and Non-Epidemic Hot Spots of Malaria Transmission Occur in Indigenous Comarcas of Panama. PLoS Negl. Trop. Dis..

[9] Instituto de Estadísticas y Censo (2021). Panamá en Cifras: Años 2015–2019.

[10] Ministerio de Ambiente y GIZ (2020). Programa de Restauración Forestal.

[11] Cecchini S., Mojica R.H.Y.A.R. (2020). La Matriz de Desigualdad Social en Panamá.

[12] Ministerio de Ambiente (2020). Plan Nacional Contra la Sequía de Panamá.

[13] CATHALAC (2016). Una Nueva Regionalización Climática de Panamá Como Aporte a la Seguridad Hídrica, Trabajo de la División de Investigación Aplicada y Desarrollo.

[14] Bortman M. (1999). Elaboración de corredores o canales endémicos mediante planillas de cálculo. Rev. Panam. Salud Pública.

[15] Venables W., Ripley B. (2002). Modern Applied Statistics with S.

[16] Borcard D., Gillet F., Legendre P. (2018). Numerical Ecology with R.

[17] Brunsdon C., Comber L. (2015). An Introduction to R for Spatial Analysis and Mapping.

[18] Kulldorff M. (1997). A spatial scan statistic. Commun. Stat. Theory Methods.

[19] Kulldorff M., Huang L., Pickle L., Duczmal L. (2006). An elliptic spatial scan statistic. Stat. Med..

[20] Kulldorff M. (2009). SaTScanTM v8.0: Software for the Spatial and Space-Time Scan Statistics.

[21] Samudio F., Santamaría A.M., Obaldía N., Pascale J.M., Bayard V., Calzada J.E. (2005). Prevalence of Plasmodium falciparum mutations associated with antimalarial drug resistance during an epidemic in Kuna Yala, Panama, Central America. Am. J. Trop. Med. Hyg..

[22] Calzada J.E., Samudio F., Bayard V., Obaldia N., de Mosca I.B., Pascale J.M. (2008). Revising antimalarial drug policy in Central America: Experience in Panama. Trans. R. Soc. Trop. Med. Hyg..

[23] Obaldia N., Baro N.K., Calzada J.E., Santamaria A.M., Daniels R., Wong W., Chang H.-H., Hamilton E.J., Arevalo-Herrera M., Herrera S. (2015). Clonal Outbreak of *Plasmodium falciparum* Infection in Eastern Panama. J. Infect. Dis..

[24] Santamaría A.M., Vásquez V., Rigg C., Moreno D., Romero L., Justo C., Chaves L.F., Saldaña A., Calzada J.E. (2020). *Plasmodium falciparum* Genetic Diversity in Panamá Based on *glurp*, *msp*-1 and *msp*-2 Genes: Implications for Malaria Elimination in Mesoamerica. Life.

[25] Santamaría A.M., Vásquez V., Rigg C., Samudio F., Moreno D., Romero L., Saldaña A., Chaves L.F., Calzada J.E. (2021). *Plasmodium vivax* Genetic Diversity in Panama: Challenges for Malaria Elimination in Mesoamerica. Pathogens.

[26] Loaiza J.R., Bermingham E., Scott M.E., Rovira J.R., Conn J.E. (2008). Species composition and distribution of adult Anopheles (Diptera: Culicidae) in Panama. J. Med. Entomol..

[27] Instituto Geográfico Nacional Tommy Guardia (2016). Atlas Nacional de la República de Panamá 2015.

[28] Servicio Nacional de Migración de Panamá (2023). Movimiento Migratorio. https://www.migracion.gob.pa/images/img2023/pdf/MOVIMIENTO_MIGRATORIO_ACTUALIZADO_DIC_2023.pdf.

[29] Naranjo L., Williams Y., Levy J., Obando R., González J.A., Pachar M., Chen R., Franco-Paredes C., Higuita N.A., Henao-Martínez A. (2023). The Endless Vulnerability of Migrant Children In-Transit across the Darién Gap. Am. J. Trop. Med. Hyg..

[30] Pacheco M.A., Forero-Peña D.A., Schneider K.A., Chavero M., Gamardo A., Figuera L., Kadakia E.R., Grillet M.E., Oliveira-Ferreira J., Escalante A.A. (2020). Malaria in Venezuela: Changes in the complexity of infection reflects the increment in transmission intensity. Malar. J..

[31] Montenegro Y.A. (2021). Sanciones impuestas por Estados Unidos a Venezuela: Consecuencias regionales. Rev. Relac. Int. Estrateg. Y Segur..

[32] Agudelo Higuita N.I., Franco-Paredes C., Henao-Martínez A.F., Mendez Rojas B., Suarez J.A., Naranjo L., Alger J. (2023). Migrants in transit across Central America and the potential spread of chloroquine resistant malaria-a call for action. Lancet Reg. Health Am..

[33] Blackburn D., Drennon M., Broussard K., Morrison A.M., Stanek D., Sarney E., Ferracci C., Huard S., Brennan W., Eaton J. (2023). Outbreak of Locally Acquired Mosquito-Transmitted (Autochthonous) Malaria—Florida and Texas, May–July 2023. MMWR. Morb. Mortal. Wkly. Rep..

[34] International Organization for Migration The Displacement Tracking Matrix (DTM). Panamá—Monitoreo de Flujo de Población Migrante—Darién y Chiriquí (Agosto 2023). https://dtm.iom.int/panama.

[35] Ministerio de Salud, Direccion General de Salud. Panamá Caracterizacion de focos malaricos. Panama 2018. https://www3.paho.org/pan/dmdocuments/Anexo%203-CARACTERIZACION-FOCOS%20MALARICOS.2019.pdf.

[36] Chaves L.F., Ramírez Rojas M., Prado M., Garcés J.L., Salas Peraza D., Marín Rodríguez R. (2020). Health policy impacts on malaria transmission in Costa Rica. Parasitology.

[37] Tatem A.J., Gething P.W., Smith D.L., Hay S.I. (2013). Urbanization and the global malaria recession. Malar. J..

