# Supplementary material for: Spatiotemporal Analysis of Malaria Transmission in the Autonomous Indigenous Regions of Panama, Central America, 2015–2022
Source: Trop Med Infect Dis. 2024 Apr 22;9(4):90. doi: 10.3390/tropicalmed9040090 (PMC11054363; doi:10.3390/tropicalmed9040090)
Supplement: Supplementary file 1 [file tropicalmed-09-00090-s001.zip › tropicalmed-2911356-supplementary.pdf]

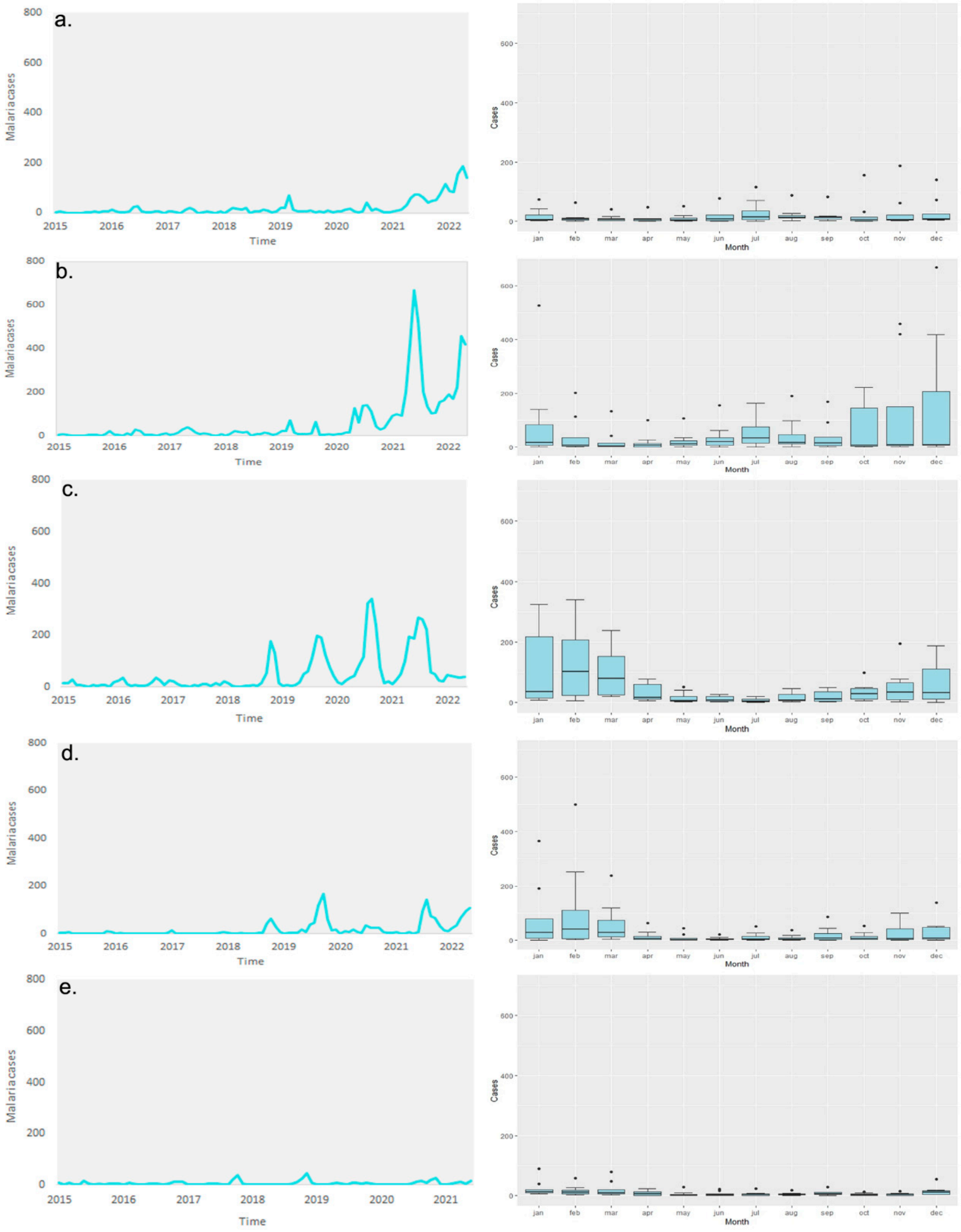

Figure S1. Weekly evolution and monthly distribution of malaria cases in each comarca from Panama, 2015-2022: a) Ngobe Bugle, b) Guna Yala, c) Madungandí, d) Wargandí, e) Emberá Wounáan.

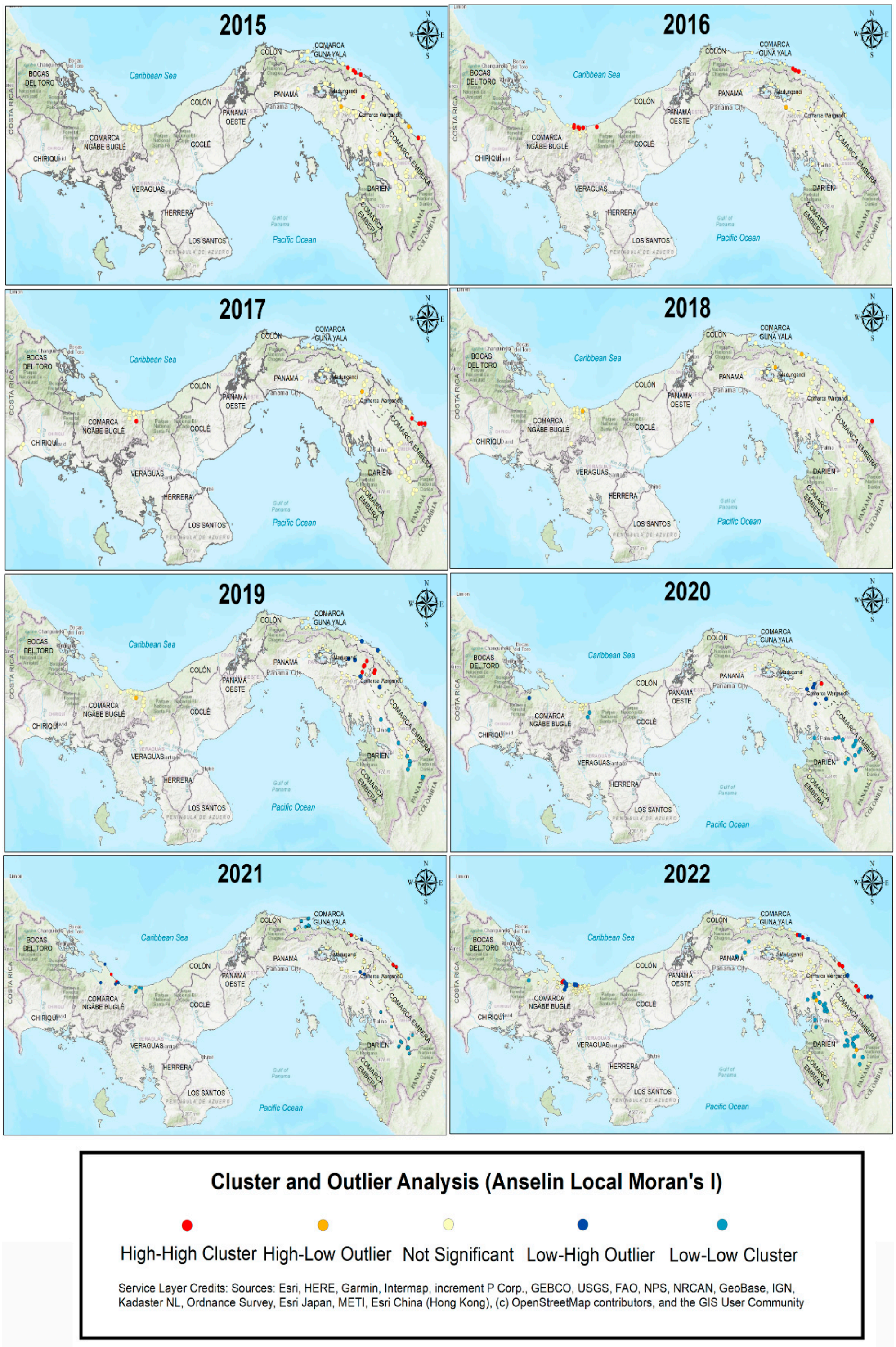

Figure S2. Spatial autocorrelation of malaria cases from 2015 to 2022 in Panamá

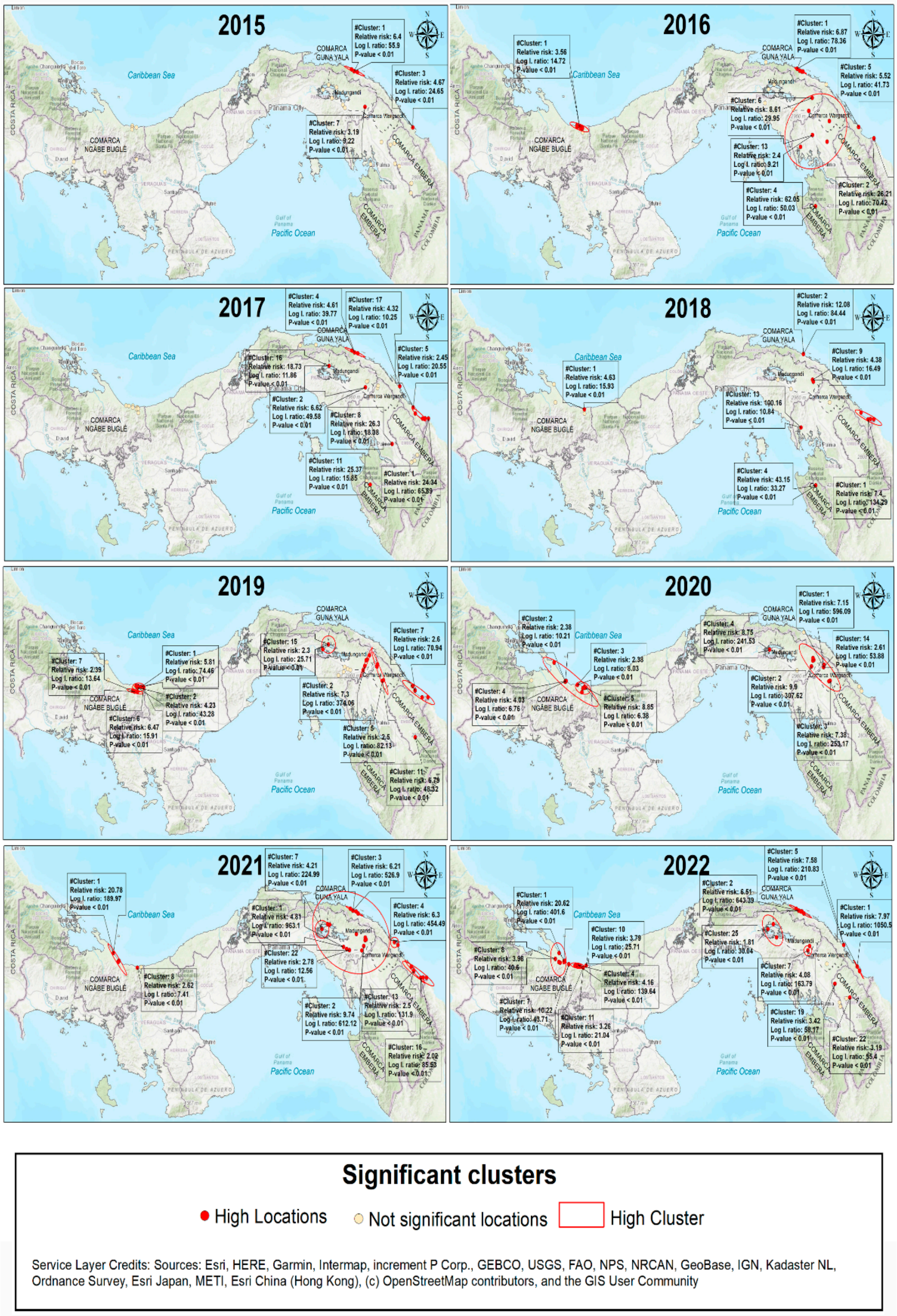

Figure S3. Cluster of malaria cases detected using the purely temporal clustering from 2015 to 2022 in Panama.

Table S1: Spatial scan statistics of significant clusters of malaria cases at the Indigenous Comarcas in the western region of Panama, January 2015 to December 2022

Year	Cluster	Locality (High clusters)	Population	Malaria cases	Malaria cases (expected)	RR	LRR	p
2016	1	Segunda Corriente	893	53	29.46	3.56	14.72	< 0.01
		Querbrada Peña,						
		Loma Chucara						
		Limoncito						
		Segunda Corriente						
		Santa Catalina Arriba						
		Santa Catalina o Calovébora						
2018	1	Aguacate Arriba	116	24	6.39	4.63	15.93	< 0.01
2019	1	Santa Catalina o Calovébora	768	166	77.88	5.81	74.46	< 0.01
		Boca de Aguacate						
		Aguacate Arriba						
		San Soledad						
		Llano Piña						
		Caño Escondido (Santa Catlina)						
		Boca de Tontri o Toncri						
		Cahuita Arriba						
		Los Primitivos						
	2	Boca de Aguacate	255	79	25.86	4.23	43.28	< 0.01
		Aguacate Arriba						
		Llano Piña						
		Caño Escondido (Santa Catalina)						
	6	Los Primitivos	26	16	2.64	6.47	15.91	< 0.01
	7	Loma Pava	278	57	28.19	2.39	13.64	< 0.01
		Alto Piña						
		San Soledad						
2020	3	Caracol No. 1	334	35	17.83	2.38	8.03	< 0.01
		Alto Piña						
		Santa Catalina Arriba or Quebrada Catalina						
		Boca de Aguacate						
		Aguacate Arriba						
		Caño Escondido						
	5	Caño Lagarto	55	11	2.94	4.03	6.76	< 0.01
2021	1	Tiger Head or Playa Tigre	151	115	12.3	20.78	189.97	< 0.01
		Librate						
		Begabudi						
		Caño Lagarto						
	7	Aguacate Arriba	116	23	9.45	2.62	7.4	< 0.01
2022	1	Librate	100	203	11.98	20.62	401.6	< 0.01
		Begabudi						
		Caño Lagarto						
	4	Chelele	561	234	67.2	4.16	139.63	< 0.01
		Caracol No. 1						
		Segunda Corriente						
		Quebrada Peña						
		Loma Chucara						
		Limoncito						
	7	Palo Blanco	26	31	3.11	10.22	43.71	< 0.01
	8	Odobari	145	66	17.37	3.98	40.6	< 0.01
		Dorori or Brodori Medio						
		Tiger Head or Playa Tigre						
		Muay Abajo						
	10	Zaragoza	100	44	11.98	3.79	25.71	< 0.01
	11	Aguacate Arriba	116	44	13.89	3.26	21.04	< 0.01

Table S2: Spatial scan statistics of significant clusters of malaria cases at the Indigenous Comarcas in the eastern region of Panama, January 2015 to December 2022.

Time	Cluster	Locality (high clusters)	Population	Malaria cases	Malaria cases (expected)	RR	LLR	p
2015	1	Achiote or Achiotal or Maguebgandi	944	78	13.26	6.87	78.36	< 0.01
		Río Diablo or Aidirgandi						
		Wichub Mullu						
		Playón Grande or Ukupa						
	3	La Mel	90	31	1.26	26.21	70.42	< 0.01
	7	Puerto Limón or Narasgandi	573	20	6.51	3.19	9.22	< 0.01
2016	1	Achiote or Achiotal or Maguebgandi	944	78	13.26	6.87	78.36	< 0.01
		Río Diablo or Aidirgandi						
		Wichub Mullu						
		Playón Grande or Ukupa						
	2	La Miel	90	31	1.26	26.21	70.42	< 0.01
	4	Boca De Tigre	19	16	0.27	62.05	50.03	< 0.01
	5	Carreto or Yansibdiwar	692	49	9.72	5.52	41.73	< 0.01
	6	Cañazas o Narganí	208	24	2.92	8.61	29.95	< 0.01
	13	La Peñita	1022	33	14.36	2.4	9.21	< 0.01
		Río Cañazas or Akanwila						
		Quebrada Muerto						
		Villa Nueva						
		Mortí						
2017	1	La Miel	90	30	1.33	24.34	65.89	< 0.01
	2	Puerto Limón or Narasgandí	573	50	8.47	6.62	49.58	< 0.01
	4	Achiote or Achiotal or Maguebgandí	943	57	13.93	4.61	39.77	< 0.01
		Río Diablo or Aidirgandí						
		Playón Grande or Ukupa						
	5	Carreto or Yansibdiwar	2406	77	35.55	2.45	20.55	< 0.01
		Anachucuna or Aswemullu						
		Armila						
		Puerto obaldía						
	8	Quebrada Grande	21	8	0.31	26.3	18.38	< 0.00
	11	Boca De Tigre	19	7	0.28	25.37	15.85	< 0.01
	16	Espavé (P)	22	6	0.33	18.73	11.86	< 0.01
	17	Isla Pino or Dubbag	242	15	3.58	4.32	10.25	< 0.01
2018	1	Anachucuna or Aswemullu	1804	139	27.22	7.4	134.29	< 0.01
		Armila						
		Puerto obaldía						
		La Miel						
	2	Playón Grande or Ukupa	354	56	5.34	12.08	84.44	< 0.01
	4	Boca de Tigre	19	12	0.29	43.15	33.27	< 0.01
	9	Cañazas or Nargandí	381	24	5.75	4.38	16.49	< 0.01
		Río Sábalo						
	13	La Peñita	3	2	0.03	100.16	10.84	< 0.01

2019	1	Río Cañazas or Akanwila	1379	371	65.84	5.64	374.06	< 0.01
		Puerto Limón or Narasgandi						
		Naca U ogobnawila						
		Rio Sabalo						
		Cañazas or Nargandi						
	5	Walá	2885	300	137.74	2.5	85.12	< 0.01
		Nurra						
		Wagandí						
		Morti						
	2	Río Cañazas or Akanwila	1379	371	65.84	7.3	374.06	< 0.01
		Puerto Limón or Narasgandi						
		Naca U ogobnawila						
		Rio Sabalo						
		Cañazas or Nargandi						
	5	Walá	2885	300	137.74	2.5	82.13	< 0.01
		Nurra						
		Wagandí						
		Morti						
	7	Carreto or Yansibdiwar	2083	232	99.45	2.6	70.94	< 0.01
		Anachucuna						
		Pueblo Nuevo-Ng						
		Armila						
		La Miel						
	11	El Tigre	146	46	6.97	6.79	48.32	
	15	Puente Bayano or Akua Yala	947	100	45.21	2.3	25.71	< 0.01
		Pintupu or Iguayala						< 0.01
2020	1	Puerto Limón or Narasgandi	4249	759	207.99	7.15	596.09	< 0.01
		Naca U ogobnawila						
		Cañazas or Nargandi						
		Río Sábalo or Kuinubdi						
		Wala						
		Nurra						
		Wagandí						
		Mortí						
	2	Puerto Limón or Narasgandi	573	234	28.05	9.9	307.62	< 0.01
	4	Akuayala or Puente Bayano	532	198	26.04	8.75	241.53	< 0.01
	14	Wala	1444	170	70.68	2.61	53.88	< 0.01

2021	1	Akuayala or Puente Bayano<br>Pintupu or Igualalla<br>Aguas Claras or Icantí<br>Ipetí Kuna<br>Wacuco or Dianwardumadí (P)<br>Río Rubén or Quidmorgandí (P)<br>Maguebgandi or Achiote<br>Aidirgandi or Piedra De Amolar<br>Playon Grande or Ukupa<br>Piria<br>Irgandi<br>Río Cañazas or Akanwila<br>Puerto Limón 0 Narasgandí<br>Naca<br>Cañazas or Nargandí<br>Río Sábalo or Kuinubdi	7397	1863	706.41	4.81	963.10	< 0.01
	2	Puerto Limón 0 Narasgandí	573	465	54.72	9.74	612.12	< 0.01
	3	Maguebgandi or Achiote<br>Aidirgandi or Piedra De Amolar<br>Playon Grande or Ukupa<br>Irgandí	1136	574	108.49	6.21	526.90	< 0.01
	4	Mansucun or Mamsuggun<br>Isla Pino	914	482	87.29	6.3	454.49	< 0.01
	7	Akuayala or Puente Bayano<br>Pintupu or Igualalla	947	350	90.44	4.21	224.99	< 0.01
	13	Yancibdiwar or Carreto<br>Anachucuna<br>Pueblo Nuevo<br>Armila	2086	456	199.21	2.5	131.90	< 0.01
	16	Yancibdiwar or Carreto<br>Anachucuna<br>Pueblo Nuevo<br>Armila<br>Puerto obaldía<br>La Miel	2689	480	256.8	2.02	85.53	< 0.01
	22	Río Rubén or Quidmorgandí (P)	125	33	11.94	2.78	12.56	< 0.01
2022	1	Carreto or Yansibdiwar<br>Anachucuna or Aswemullu<br>Pueblo Nuevo	1441	958	151.34	7.97	1050.59	< 0.01
	2	Maguebgandi or Achiote or Achiotal<br>Airdirgandí or Piedra De Amolar<br>Wichub Mullu<br>Playon Grande or Ukupa<br>Irgandi	1137	669	119.41	6.51	643.39	< 0.01
	5	Isla Pino	242	185	25.42	7.58	210.83	< 0.01
	7	Río Cañazas or Akanwila<br>Puerto Limon or Narasgandi	635	259	66.69	4.08	163.79	< 0.01
	19	Canaán Membrillo<br>Río Membrillo	321	113	33.71	3.42	58.17	< 0.01
	22	Bajo Chiquito	363	119	38.12	3.19	5.40	< 0.01
	25	Akuayala or Puente Bayano<br>Pintupu or Iguayala<br>Wagandi or Wuagandi	1146	213	120.36	1.81	30.04	< 0.01
